# A phylogenetic study of *Drosophila* splicing assembly chaperone RNP-4F associated U4-/U6-snRNA secondary structure

**DOI:** 10.4236/ojas.2013.34A2005

**Published:** 2013-11

**Authors:** Jack C. Vaughn, Sushmita Ghosh, Jing Chen

**Affiliations:** Department of Biology, Cell Molecular and Structural Biology Program, Miami University, Oxford, USA

**Keywords:** RNP-4F, snRNA Secondary Structure, U4-/U6-snRNA Phylogeny, Spliceosome Evolution

## Abstract

The *rnp*-4*f* gene in *Drosophila melanogaster* encodes nuclear protein RNP-4F. This encoded protein is represented by homologs in other eukaryotic species, where it has been shown to function as an intron splicing assembly factor. Here, RNP-4F is believed to initially bind to a recognition sequence on U6-snRNA, serving as a chaperone to facilitate its association with U4-snRNA by intermolecular hydrogen bonding. RNA conformations are a key factor in spliceosome function, so that elucidation of changing secondary structures for interacting snRNAs is a subject of considerable interest and importance. Among the five snRNAs which participate in removal of spliceosomal introns, there is a growing consensus that U6-snRNA is the most structurally dynamic and may constitute the catalytic core. Previous studies by others have generated potential secondary structures for free U4- and U6-snRNAs, including the Y-shaped U4-/U6-snRNA model. These models were based on study of RNAs from relatively few species, and the popular Y-shaped model remains to be systematically re-examined with reference to the many new sequences generated by recent genomic sequencing projects. We have utilized a comparative phylogenetic approach on 60 diverse eukaryotic species, which resulted in a revised and improved U4-/U6-snRNA secondary structure. This general model is supported by observation of abundant compensatory base mutations in every stem, and incorporates more of the nucleotides into base-paired associations than in previous models, thus being more energetically stable. We have extensively sampled the eukaryotic phylogenetic tree to its deepest roots, but did not find genes potentially encoding either U4- or U6-snRNA in the *Giardia* and *Trichomonas* data-bases. Our results support the hypothesis that nuclear introns in these most deeply rooted eukaryotes may represent evolutionary intermediates, sharing characteristics of both group II and spliceosomal introns. An unexpected result of this study was discovery of a potential competitive binding site for *Drosophila* splicing assembly factor RNP-4F to a 5’-UTR regulatory region within its own premRNA, which may play a role in negative feedback control.

## 1. INTRODUCTION

*Drosophila melanogaster*, which is a cosmopolitan holometabolous insect found in all warm environments, has been an important model organism for genetic, molecular, cellular and physiological studies for over a century. Its small size (usually 2 – 4 mm), short life cycle (10 – 14 days at 25°C), high reproductive rate (an adult female can lay 400 – 500 eggs in 10 days), completely sequenced and largely annotated genome, well-developed techniques, and evolutionarily-conserved molecular pathways all contribute to make *Drosophila* a research paradigm. It has been predicted that about 75% of human disease genes have clear homologs in *D. melanogaster* [1,2], an observation leading to the extensive use of *Drosophila* which has led to advances in the improvement of human health.

The long-term objective of our research is to understand evolutionarily-conserved cellular, developmental, molecular and genetic mechanisms behind regulation of genes which encode intron splicing assembly factor proteins, a topic about which relatively little is known. The system which we are currently using to address these questions is the *Drosophila rnp*-4*f* gene, which encodes splicing assembly factor RNP-4F, and we are concentrating on mechanisms of posttranscriptional level regulation [3–11]. This protein is believed to play a direct role during spliceosome assembly by acting as a chaperone to unwind U6-snRNA and thus facilitate its association with U4-snRNA *via* intermolecular hydrogen bonding [12–16]. In the course of our work, we became interested in secondary structure interactions within the *Drosophila* U4-/U6-snRNA duplex.

The major or U2-type molecular pathway for removal of spliceosomal introns has been extensively studied [reviewed in 17,18], and shown to require direct participation of five *trans*-acting small nuclear uracil-rich RNAs (snRNAs) termed U1, U2, U4, U5 and U6. These RNAs are each associated with specific sets of proteins to yield the corresponding biologically active snRNPs, which progressively interact with pre-mRNAs and with each other during the ensuing spliceosomal assembly. In addition to these snRNAs, about 70 different snRNP proteins and more than 100 non-snRNP proteins have been shown to be spliceosomal components [reviewed in 19]. For example, the essential *Saccharomyces cerevisiae* pre-mRNA splicing protein Prp24, represented in *Drosophila* by its ortholog RNP-4F and in human by p110 [13,14] facilitates U4- and U6-snRNA pairing during spliceosomal assembly [16].

A succession of snRNA conformational changes accompanies steps in the splicing pathway, which are essential in generation and function of the catalytic structure. Elucidation of the changing secondary structures of the interacting snRNA molecules is therefore a subject of considerable interest and importance. The comparative phylogenetic approach [20,21] generates models in which existence of potential biologically significant stem-loops can be established by observation of compensatory base mutations in diverse species, and has proven to be a powerful technique. The original Y-shaped U4-/U6-snRNA duplex secondary structure model [12] was based on this methodology by comparing yeast, fruit-fly, plant and human sequences. Subsequent studies have shown that RNAs from various species can also be folded in accordance with this model [22–26]. However, no attempt has ever been made to systematically re-examine the original model itself, utilizing the relative abundance of new sequences now available for analysis.

## 2. MATERIALS AND METHODS

### 2.1. Selection of U4- and U6-snRNA Sequences

We began by utilizing the original Small RNA Database [27] as a source for sequences published early. We then carried out GenBank searches, followed by BLAST searches (http://www.ncbi.nlm.nih.gov/BLAST) in which bait sequences were derived from the major phylogenetic levels. Finally, the number of sequences available for study was further increased from early published work not submitted to GenBank. The BLAST search was more successful in finding U6-snRNAs, owing to their extremely high sequence conservation. We did not use every sequence found, excluding for example those from eleven other *Drosophila* species [28] and also different species of *Saccharomyces*, since their inclusion would add little additional understanding due to having virtually identical sequences within a genus. This exercise (Table 1) yielded 42 U4- and 56 U6-snRNAs, of which 38 were both available in a given species and deemed optimal for our study. In total, sequences from some 60 different species were included in our study.

### 2.2. Alignment of U4- and U6-snRNA Sequences

All sequences selected for this study were individually aligned with reference to the corresponding *Drosophila* genes using the ClustalW program (http://align.genome.jp), and the resulting alignment was further refined by eye. Finally, the alignment was adjusted using the emerging secondary structure results, to assure that homologous nucleotides would be compared for evidences of compensatory base mutations. The final alignments (not shown) included as few deletions (gaps) and insertions as possible, while generating the maximum number of matching residues.

### 2.3. Strategy for U4-/U6-snRNA Duplex Secondary Structure Determination

We elected to start completely from the beginning in deriving our secondary structure model, in contrast to merely modifying existing models, to optimize the chances of identifying structural components not previously recognized. We began by utilizing version 3.6 of the Mfold program (http://mfold.rna.albany.edu) [29] for the two genes individually from *Drosophila melanogaster* (fruit-fly), *Homo sapiens* (human), *Arabidopsis thaliana* (plant), *Kluyveromyces lactis* (yeast) and *Trypanosoma brucei* (flagellate). GenBank accession numbers are given in Table 1. These structures contained a variety of potential stem-loops, and were combined to include only stem-loops held in common. The resulting U4- and U6-snRNA structures were then combined to accommodate base-pairing between the two molecules in the two closely adjacent U6 locations previously determined by photochemical cross-linking in mammalian snRNAs [30] and by subsequent observation of compensatory base mutations [12], which resulted in further simplification of potential stem-loops in the predicted duplex RNA structure. Compensatory base changes were then entered onto the *Drosophila* duplex structure in comparison with the five species originally used to begin the study (above), using the alignment to assure that homologous nucleotides were being compared. We adopted the criterion [20] that existence of a helix is considered proven if there are at least two base-pair replacements. Stems as short as two base-pairs are acceptable if compensatory base changes can be demonstrated (Carl Woese, personal communication). Finally, the provisional model was compared to every species utilized in the study (Table 1), to determine the extent to which the resulting structure was universal. When an otherwise proven stem-loop was found to be absent from any taxonomic level, the timing of that loss was charted with reference to the eukaryotic phylogenetic tree [31].

## 3. RESULTS AND DISCUSSION

### 3.1. An Improved General Secondary Structure Model for U4-/U6-snRNA

**Figure 1:**
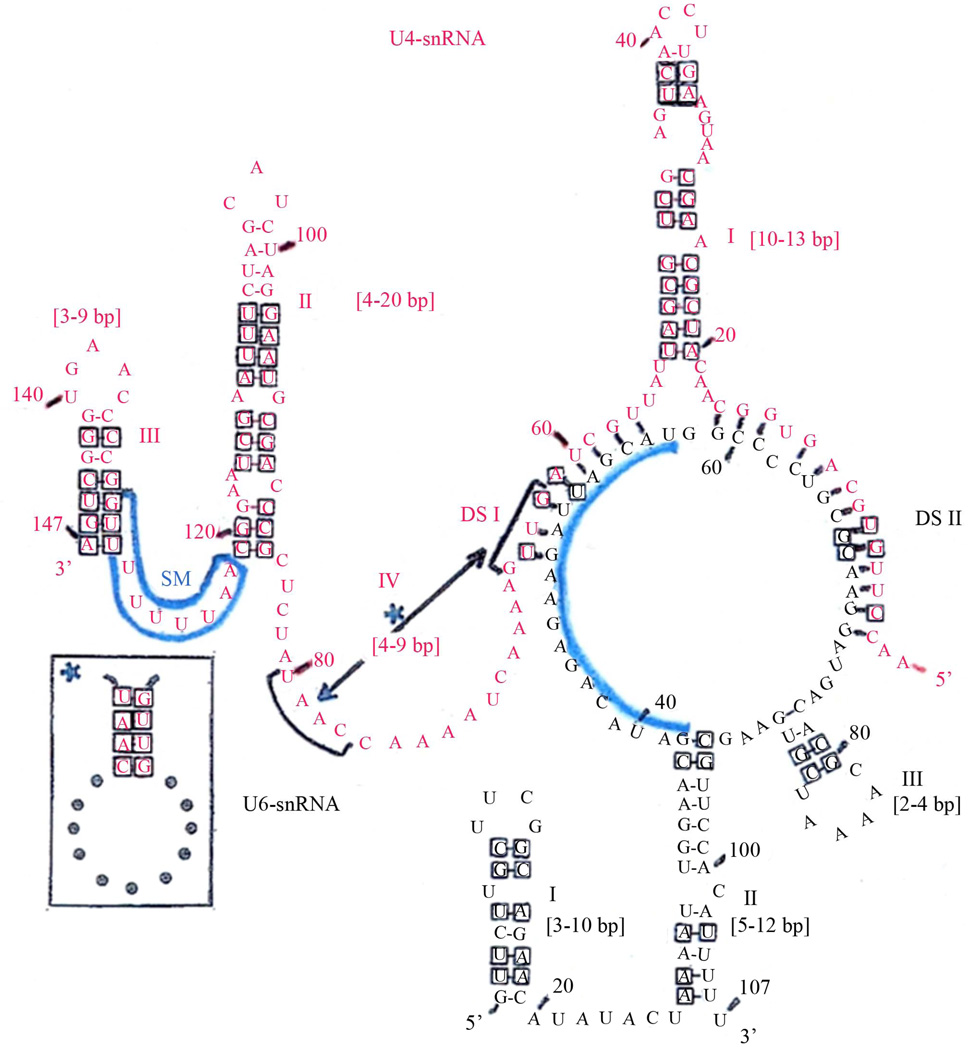
General secondary structure model for *Drosophila* U4-/U6-snRNA duplex. The two RNAs interact by base-pairing within regions designated DS I and DS II. Compensatory base changes which prove the structure illustrated are boxed and were identified in the alignment with reference to the structures derived for *H. sapiens*, *A. thaliana*, *K. lactis* and *T. brucei*. The range of stem lengths found between different species in our study is shown beside each stem. Stem-loop IV in free U4-snRNA (large box) is disrupted upon binding to U6-snRNA. The putative SM-binding site (SM) is indicated. An RNA recognition motif (RRM) in chaperone RNP-4F/Prp24/p110 binds primarily to a tract within free U6-snRNA nucleotides #38–57 (13), which is indicated by a heavy vertical overlay.

**Figure 2:**
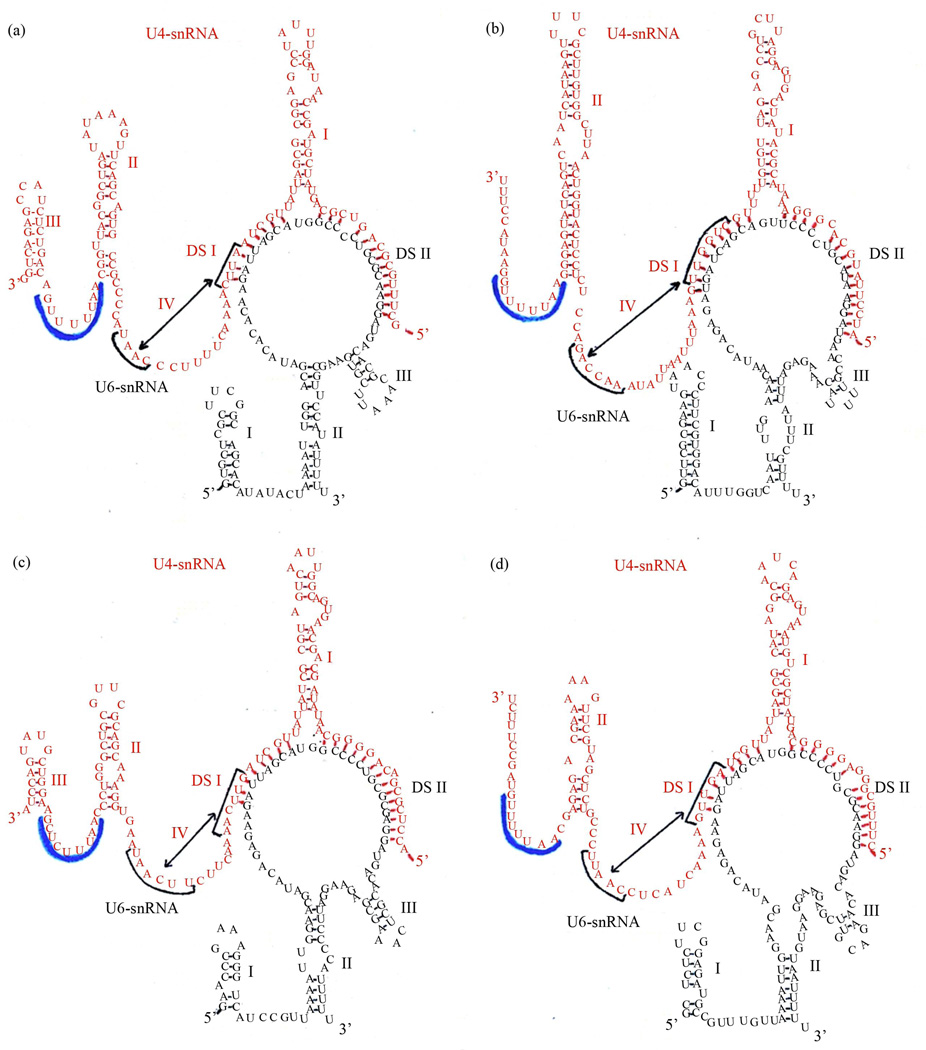

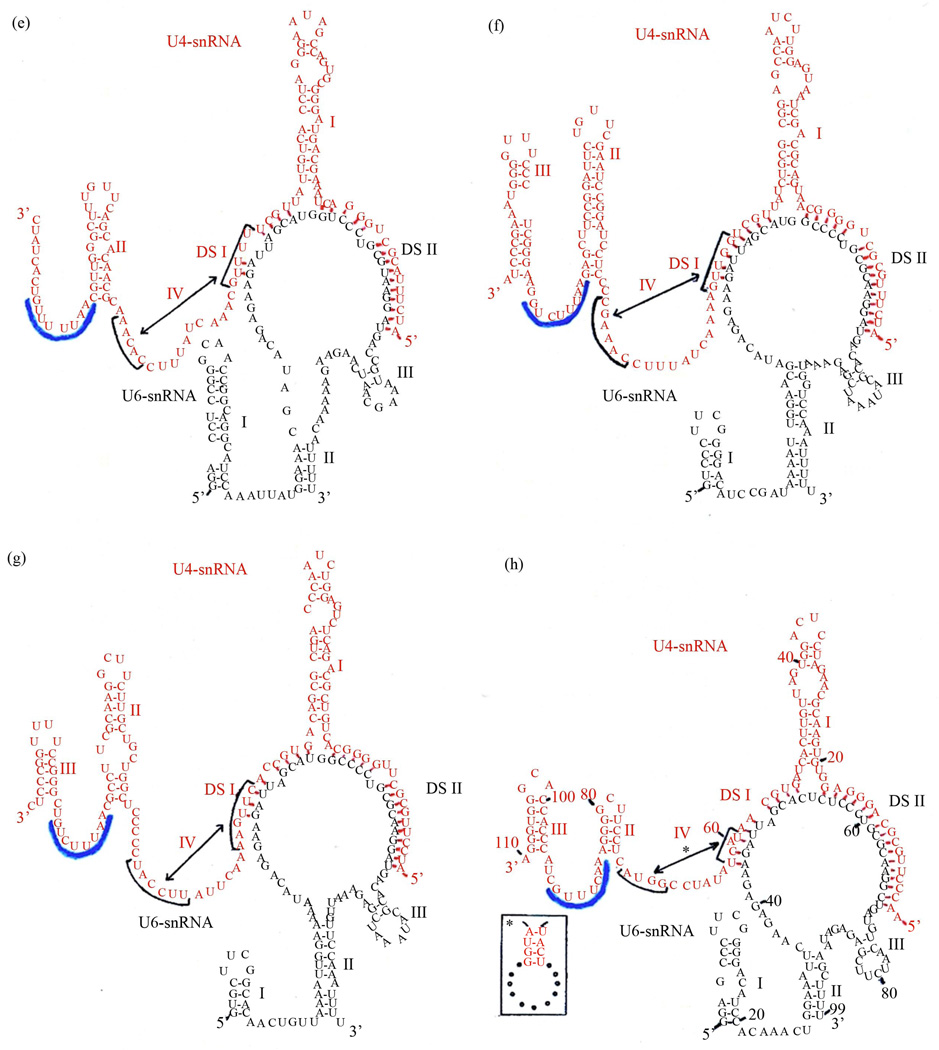
Representative U4-/U6-snRNA secondary structures from phylogenetically diverse species, folded according to our general model. (a) *H. sapiens*; (b) *S. cerevisiae*; (c) *T. thermophila*; (d) *P. falciparum*; (e) *D. discoideum*; (f) *A. thaliana*; (g) *C. reinhardtii*; (h) *T. brucei*. Labeling is as in Figure 1.

The derived U4-/U6-snRNA duplex secondary structure model is shown in Figure 1, and structures from representative species at different taxonomic levels in Figures 2(a)–(h). A relatively large proportion of all nucleotides are base-paired in our U4-/U6-snRNA model. For example, in *Drosophila* 58% are base-paired in U4 and 63% in U6, whereas in the Y-shaped model the corresponding numbers are 58% and 33%. Four stem-loops (I-IV) are found to be present in the U4 structure for most species, so that our model both confirms and extends the secondary structure for free U4-snRNA previously proposed [32] using the phylogenetic approach with far fewer species. The existence of stem-loop IV in free U4-snRNA, proposed by the same authors, is also confirmed for all species studied by us. The overall conformation of the structure shown in our model is very similar in every species examined, with the exception of stem-loop III in U4-snRNA which is further discussed in Section 3.2. Each stem in our model has been proven by observation of numerous compensatory base mutations. Species within the flagellate group Euglenozoa were found to have the shortest overall U4- and U6-snRNA lengths (compare *D. melanogaster* in Figure 1 with *T. brucei* in Figure 2(h)). Despite the close similarity in conformation among species, nearly all stem lengths are however quite variable (Figure 1). The most consistent stem length is in U4 stem I, which ranges from 10 – 13 base pairs and is always interrupted by a structurally conserved bulge loop. A conspicuous highly conserved sequence tract in U4 is the putative SM-binding site, located near the 3’-end between stem-loops II and III, which matches the consensus sequence AU [4–6] G. Our study confirms the universality of the two major intermolecular base-paired zones of contact between the two RNA molecules (DS I and DS II) as originally proposed [12], with many examples of compensatory base mutations.

The U6-snRNA nucleotide sequence is relatively highly conserved, in comparison with that for U4. Three stem-loops are also present in the U6 structure in our duplex model, which is in contrast to the Y-shaped model in which only stem-loop I is shown. In our model the 3’-end of U6-snRNA is incorporated into the structure to form stem-loop II in every species examined, albeit in some cases with a central bulge loop or absence of base-pairing at the top of the stem. U6 stem-loop II is proven by observation of compensatory base mutations, and is not shown in other models. A second U6 structural feature in our model which is not shown in other models is a short stem-loop III. This stem-loop is only two base-pairs long in many species, but is proven by observation of compensatory base mutations. We did however fail to observe this stem-loop in the fungus *C. albicans* and in *E. histolytica*, showing that it is not universal.

### 3.2. The General Secondary Structure Model is Not Universal and Multiple Independent U4-snRNA Stem-Loop III Losses Have Occurred During Evolution

**Figure 3:**
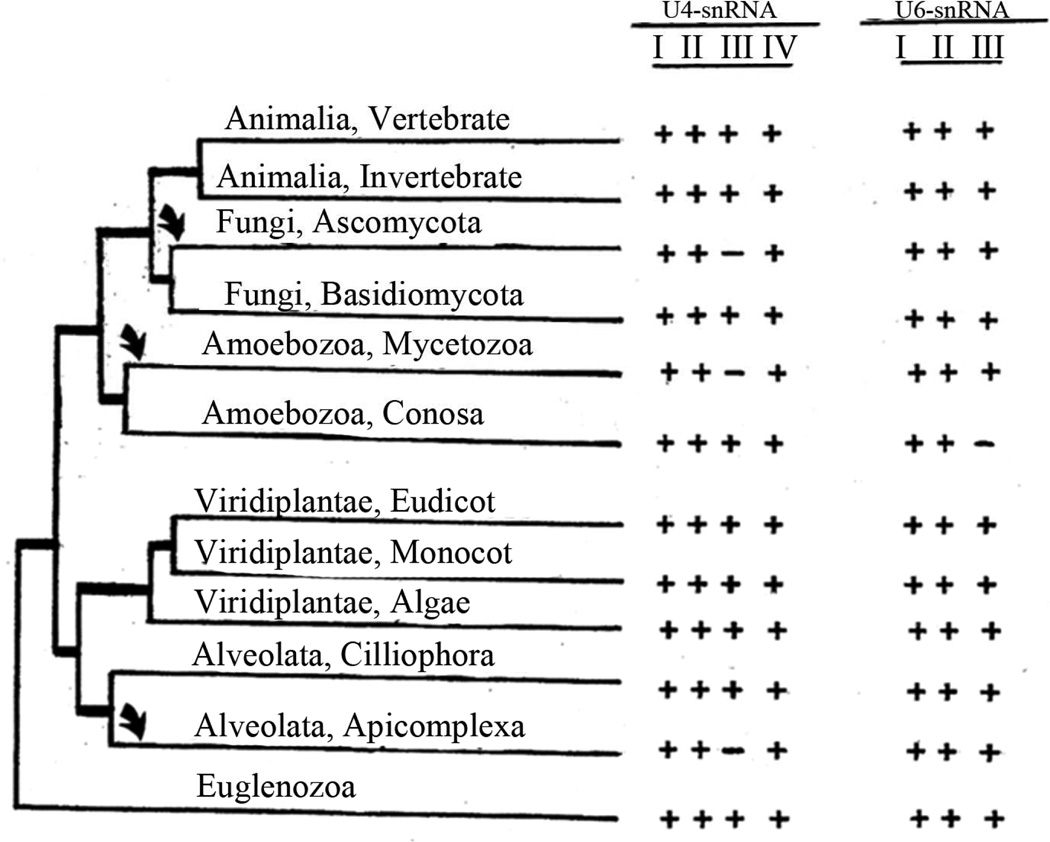
Eukaryotic phylogenetic tree (31), showing taxonomic distribution of species included in our study and stem-loops observed. U4-snRNA stem-loop III has been independently lost at least three times (arrows) during evolution of these RNAs.

Representative structures for a diverse selection of evolutionarily distant species show that the general model is not universal. The most striking example is in the absence of U4-snRNA stem-loop III (Figures 2(b), (d), (e)), otherwise proven by observation of numerous compensatory base changes. The absence of this stem-loop has previously been noted in secondary structures for various species of yeast and slime molds [12,25,32,33]. It has been suggested that the absence of this stem-loop is correlated with phylogenetic depth, implying that this structural feature was not present in the earliest eukaryotes and is newly evolved [32]. We tested this hypothesis by superimposing the presence/absence of this stem-loop onto the eukaryotic phylogenetic tree [31]. The results show that this stem-loop is present in all species among the deeply-rooted flagellate Euglenozoa examined, but that three clearly independent secondary losses have occurred during evolution (Figure 3). The most recent is within Fungi, where all Ascomycete species studied have lost the stem-loop, which is however present in the Basidiomycete *E. hasegawianum*. An earlier independent loss occurred among the Amoebozoa, where the Mycetozoa slime mold species examined have lost the stem-loop but the amoeboid Conosa *E. histolytica* has not. The earliest loss is in the Alveolata, where this feature is absent in the Apicomplexa *P. falciparum* but not in the Cilliophora *T. thermophila*.

### 3.3. The General Secondary Structure Model Compared to the Classical Y-Shaped Model

**Figure 4:**
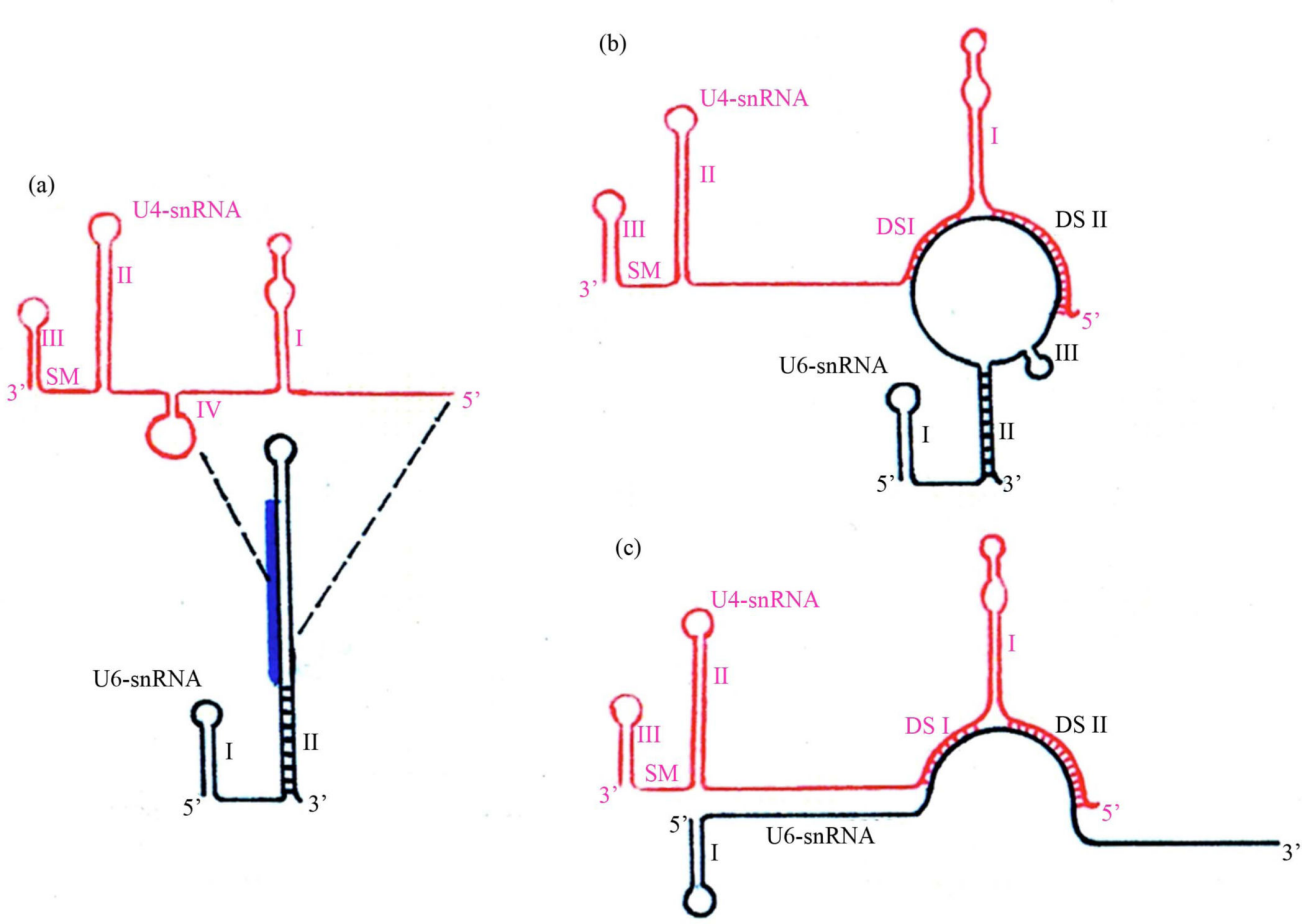
Comparison between our general U4-/U6-snRNA secondary structure and the Y-shaped model. (a) Structures of free U4-(32) and U6-snRNA (35) prior to their interaction. The primary position for binding of RRM in chaperone RNP-4F/Prp24/p110 to free U6 stem-loop II (13) is indicated by heavy vertical overlay, and was determined experimentally. The unwinding of U6 stem-loop II due to chaperone activity permits base-pairing between the two RNAs (region bounded by the broken lines). The base of stem-loop II (cross-bars) remains associated in the resulting duplex structure in our model. (b) Our general secondary structure model. (c) The Y-shaped model (12), shown inverted to facilitate comparisons.

It is informative to compare the secondary structures of free U4- and U6-snRNAs with that of the duplex which is formed upon their association during spliceosome assembly, in consideration of the most parsimonious solution for their association (Figure 4). An excellent free U4-snRNA secondary structure model has previously been proposed based on the phylogenetic approach [32], utilizing a taxonomic diversity of species extending only as deep as the slime mold *Physarum*. This structure has been experimentally supported by the results of enzymatic digestion studies in rat U4-snRNA [34]. The model contains four stem-loops, of which three are incorporated directly into both our model and the Y-shaped model. Stem-loop IV is disrupted in favor of intermolecular base-pairing to form DS I, upon association with U6-snRNA. Our results confirm and extend the previously proposed free U4-snRNA model, showing that the structure has been retained to its origin within the flagellate group Euglenozoa (Figure 3). There are no differences in this part of our model in comparison to the Y-shaped model.

Previously proposed free U6-snRNA models for human [35] and yeast *S. cerevisiae* [36] show somewhat differing structures, which are both supported by the results of chemical and enzymatic probing in these species [37]. In the simplest free U6-snRNA secondary structure model, as exemplified in *Drosophila* (Figure 1) and human, a short stem-loop is present at the 5’-end and the entire 3’-terminus is folded into one long interrupted stem-loop (Figure 4(a)). In human the chaperone p110, an ortholog of *Drosophila* RNP-4F, has been shown to bind primarily to free U6-snRNA nucleotides #38–57 [13], promoting unwinding of the long stem-loop and base-pairing to two closely adjacent tracts on U4-snRNA, which we have designated as DS I and DS II, followed by chaperone release.

Our model and the Y-shaped model differ primarily in how they show the U6-snRNA structure within the RNA duplex. In the latter model, only the 5’-end stem-loop is retained (Figure 4(c)), and no base-pairing occurs elsewhere except within regions DS I and DS II, so that the 3’-end is unpaired. In our model, the base of old free U6-snRNA is retained in stem-loop II, which brings the 3’-end into a duplex structure (Figure 4(b)). One set of observations in support of this structure is seen in the compensatory mutations present in this stem (Figure 1). The results of previously reported chemical and enzymatic probing of the U4-/U6-snRNA duplex further support the model which we have proposed and not the Y-shaped model. In human, chemical reagent modifications were not observed within nucleotides #27–38 or #94–106, which comprise the helix in stem-loop II in our model but which are shown in long unpaired 5’- and 3’-tracts in the Y-shaped model. These observations are indicative of a double-stranded structure here, and this interpretation is confirmed by the observation of RNase V_1_ cleavage 3’ to positions 33 and also 35 in the human U4-/U6-snRNA duplex [37]. This is an enzyme which cleaves specifically double-stranded RNA regions. These results have also been reported by these authors upon probing the base of free U6-snRNA stem-loop II. It has been proposed that a potential third base-paired region of contact may exist between U4- and U6-snRNA [24]. In this view, the top of our U6-snRNA stem-loop II is base-paired with a complement located within the long single-stranded U4 connective between DS I and U4 stem-loop II. We are skeptical of this proposed third zone of RNA/RNA interaction, since different nucleotides in the alignment must be utilized to create this structure. For example, in *S. cerevisiae* and *K. lactis* the U4 region of contact is very different from that for other species.

Within free U6-snRNA, nucleotides comprising stem-loop III are contained within the long stem-loop (Figure 4(a)). The existence of stem-loop III in the duplex structure is proven by observation of compensatory base mutations, but the stem length is reduced to only two base-pairs in many species. Chemical and enzymatic probing of the human U4-/U6-snRNA duplex [37] did not provide any further clarification for existence of this stem-loop, since most of this region was contained in the site of the primer utilized. Cryo-electron microscopy of isolated U4-/U6-snRNA has been reported to show two major structural domains linked by a thin connective [38], in good agreement with our general secondary structure model.

### 3.4. Phylogenetic Depth of the Genes Encoding U4- and U6-snRNAs

The secondary structure of the U4-/U6-snRNA duplex in our model is found to be identical, with the exception of multiple independent losses of U4 stem-loop III discussed above, down to and including the deeply-rooted flagellate group Euglenozoa. However, extensive BLAST searches against both the *Giardia* [39] and *Trichomonas* [40] genome sequences failed to detect any U4- or U6-snRNA orthologs, using the corresponding *T. brucei* sequences as bait. The diplomonads and parabasalids are generally considered to be descendants of the earliest extant eukaryotes [31], leading us to consider the implications of this observation.

Success in BLAST searches is dependent on the degree of nucleotide conservation between bait and prey sequences, in addition to the completeness and accuracy of the genomic sequence database itself. The nucleotide sequences of genes encoding U6-snRNAs are among the most highly conserved of any eukaryotic genes. For example, the human and *Drosophila* U6-snRNA sequences are 94% identical. The U6-snRNA sequence within and immediately flanking the region of base-pairing with U4-snRNA is exceptionally well conserved. For example, comparison between *Drosophila* and flagellate *T. brucei* U6 nucleotides #40–75 shows 86% identity. This degree of conservation is far greater than that observed for U4, making identification of its most ancient orthologs more difficult. It was therefore surprising that no U6-snRNA genes turned up during BLAST searches against both the diplomonad and parabasalid genomes.

The *Giardia* and *Trichomonas* genome annotations are well along, and we therefore asked if ANY of the U-series snRNA gene sequences have been annotated in these species. Surprisingly, NONE of these genes have been found despite an ~7X coverage during sequencing. In addition, none of the genes encoding proteins which are part of the U4- and U6-snRNPs in other eukaryotes have been found (Steven Sullivan, personal communication). Annotation of the *Giardia* genome has also failed to detect any genes encoding U4- or U6-snRNA (Hilary Morrison, personal communication). What are the implications of these observations? The spliceosome is widely viewed as having evolved from self-splicing group II introns like those in organellar protein-encoding genes as well as in many bacteria [reviewed in 41,42], which do not utilize the U-series of snRNAs. Interestingly, it has been proposed that *Giardia* and *Trichomonas* nuclear introns may represent evolutionary intermediates, showing characteristics of both group II and spliceosomal introns [43]. If so, then our study suggests that genes encoding U4- and U6-snRNAs, and the resultant duplex RNA which forms between them with a virtually identical secondary structure among all eukaryotes, may have evolved within the flagellate group Euglenozoa.

### 3.5. A Potential Secondary RNP-4F Chaperone Recognition Site in the 5’-UTR of *Drosophila rnp-4f* Pre-mRNA May Play a Key Role in Controlling Its Own Expression

**Figure 5:**
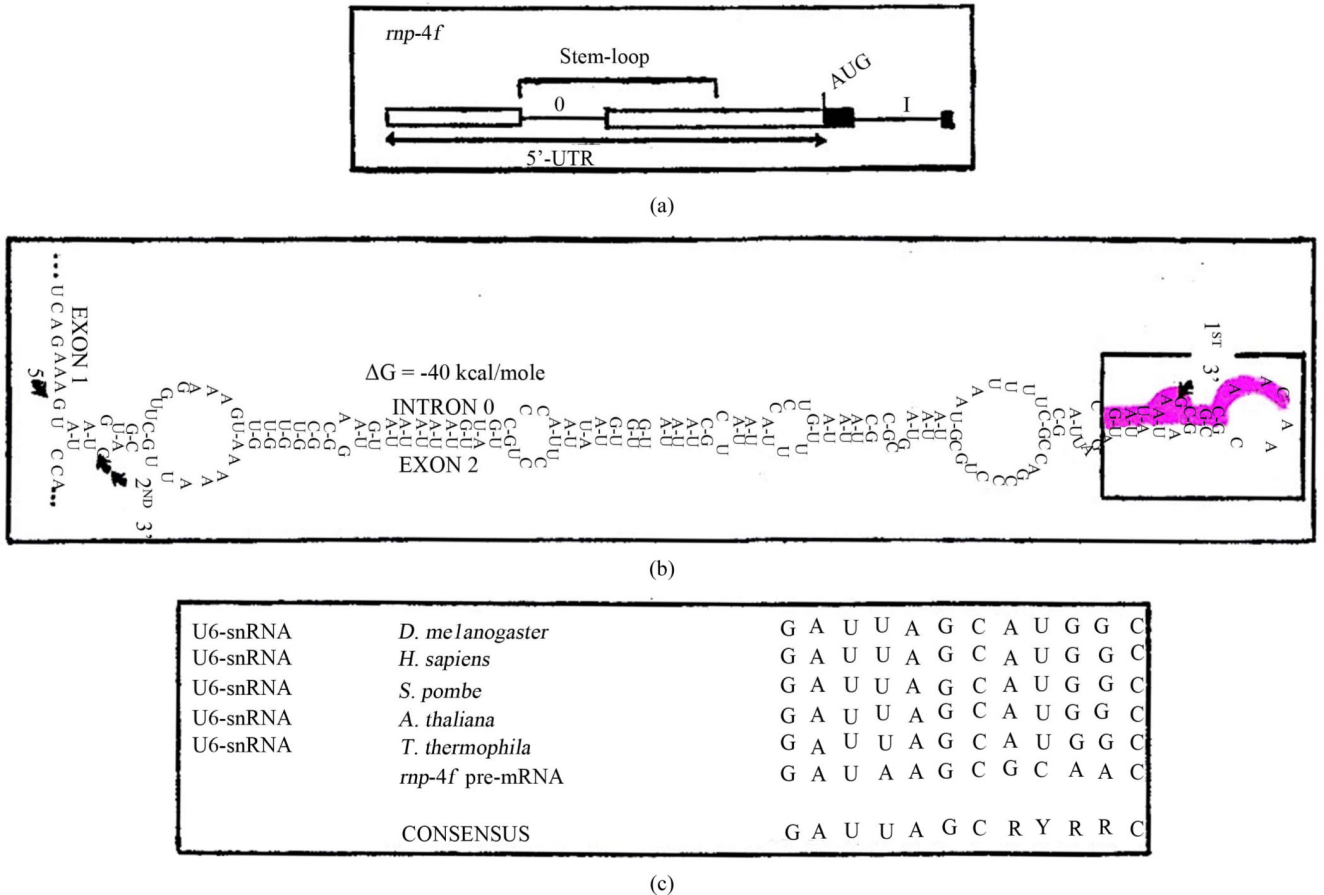
A 177-nt long *Drosophila rnp*-4*f* stem-loop in the pre-mRNA 5’-UTR regulatory region contains a potential RNP-4F protein chaperone binding site. (a) Orientation diagram showing position of long stem-loop which forms by hydrogen bonding between intron 0 and part of exon 2. (b) Long interrupted *rnp*-4*f* stem-loop secondary structure as predicted from Mfold program (29). The 5’- and 3’-limits of intron 0 are indicated, in addition to alternative 3’-splice site within exon 2 (8) and evolutionarily-conserved short stem-loop (boxed) at tip of the longer structure (6). The highlighted nucleotides near the tip show position of potential RNP-4F protein binding site postulated to compete with the preferred experimentally determined tract within U6-snRNA (13). (c) Alignment at region of chaperone RNP-4F/Prp24/p110 binding site to U6-snRNA in various species, and to potential *rnp*-4*f* pre-mRNA nucleotides.

We have previously described a long evolutionarily-conserved potential stem-loop which arises by base-pairing between all of the *rnp*-4*f* pre-mRNA intron 0 and part of adjacent exon 2 in *D. melanogaster* [6,8]. We have recently shown using RNA electrophoretic mobility shift assay that retention of intron 0 within the *rnp*-4*f* 5’-UTR is correlated with binding of a dADAR protein isoform, and that an unidentified second protein suspected to be RNP-4F also binds to this stem-loop [9]. Subsequent work employing RNAi technology showed that this dADAR protein is the truncated isoform [11]. We have proposed a negative feedback model for regulating expression of *rnp*-4*f* mRNA under conditions of RNP-4F excess within the developing fly central nervous system [6]. If this hypothesis is correct, then the conserved long stem-loop would be expected to contain a nucleotide recognition sequence to which RNP-4F could potentially bind, in competition with its preferred binding to a conserved sequence tract within the long stem-loop of free U6-snRNA [13]. In *Drosop*hila U6-snRNA the conserved sequence contains nucleotides between positions #38–57, although an even shorter sequence may suffice for chaperone binding, but this possibility has not yet been tested. Examination of the *Drosophila* conserved *rnp*-4*f* 177-nt stem-loop nucleotide sequence/structure shows that a 12-nt tract closely resembling the preferred U6-snRNA binding site is indeed present (Figure 5(b), (c)). An additional similarity between the RNP-4F chaperone substrate free U6-snRNA (Figure 4(a)) and *rnp*-4*f* pre-mRNA is that in both cases the recognition sequence is contained within a long, interrupted stem-loop structure. In *Drosophila* free U6-snRNA this stem-loop contains 81-nt, while in *rnp*-4*f* the stem-loop contains 177-nt. Finally, RNP-4F is a nuclear protein (6) and thus would be expected to have access to the long stem-loop in *rnp*-4*f* pre-mRNA. These observations support the hypothesis that excess RNP-4F protein may competitively bind to a 5’-UTR regulatory region within its own pre-mRNA, playing a role in negative feedback control.

## 4. CONCLUSIONS

Our long standing interest in *Drosophila* splicing assembly factor RNP-4F, which functions as a chaperone to facilitate bonding between U4- and U6-snRNA, led us to analyze the secondary structure of the U4-/U6-snRNA duplex. Close study of published chemical and enzymatic probing results on the proposed human and yeast *S. cerevisiae* U4-/U6-snRNA structures [37] suggested to us certain inconsistencies within the classical Y-shaped model [12]. Further, preliminary comparison of the classical model with a computer-generated secondary structure also revealed inconsistencies, which led us to reexamine this model. We deemed this timely in light of the many new U4-and U6-snRNA sequences that have become available, in large part, by recent genomic sequencing projects. Our study, utilizing the comparative phylogenetic approach, eventually resulted in a revised and improved U4-/U6-snRNA secondary structure model. The model is proven by observation of abundant compensatory base mutations in every stem, is shown to be general but not universal, and structural variations have been traced to their origins within the phylogenetic tree. We have extensively probed the eukaryotic tree to its deepest roots, and our results suggest that U4- and U6-snRNAs apparently evolved after the emergence of lines leading to the diplomonad *Giardia* and the parabasalid *Trichomonas*, but once established have maintained a remarkably well conserved U4-/U6-snRNA secondary structure extending to, and including, the flagellates among the Euglenozoa. An unexpected result of this study was discovery of a potential competitive binding site for *Drosophila* splicing assembly factor RNP-4F to a 5’-UTR regulatory region within its own pre-mRNA, which may play a role in negative feedback control [6]. This negative feedback expression control model awaits experimental testing.

## Figures and Tables

**Table l:** U4 and U6 RNA sequences utilized in this study.

Organism	GenBank Accession Number or Reference	
	U6-snRNA	U4-snRNA
**Animalia, Vertebrate**		
*Homo sapiens* (human)	X07425	X59361
*Pan troglodytes* (chimpanzee)	AC146131	NW_001223167
*Macaca mulatta* (monkey)	NW_001218112	NW_001096649
*Mus musculus* (mouse)	X06980	AC159539
*Rattus norvegicus* (rat)	AC120800	K00477
*Canis familiaris* (dog)	AC188530	NW_876282
*Bos taurus* (cattle)	NW_001492849	NW_001493540
*Sus scrofa* (pig)	CR956385	-----
*Equus caballus* (horse)	NW_001799704	NW_001799734
*Monodelphis domestica* (opossum)	NW_001581906	NW_001584232
*Ornithorhynchus anatinus* (duck-billed platypus)	NW_001794177	NW_001765942
*Gallus gallus* (chicken)	NW_001471627	M14136
*Xenopus tropicalis* (frog)	M31687	-----
*Danio rerio* (zebrafish)	CU466287	NW_001514552
**Animalia, Invertebrate**		
*Drosophila melanogaster* (fruit-fly)	X06669	D00043
*Aedes aegypti* (mosquito)	AAGE02013372	-----
*Anopheles gambiae* (mosquito)	NZ_AAAB02008807	-----
*Culex pipiens* (mosquito)	AAWU01008690	AAWU01009244
*Apis mellifera* (honey bee)	NW_001253045	-----
*Nasonia vitripennis* (jewel wasp)	NW_001815737	AAZX01001234
*Bombyx mori* (silkworm moth)	AADK01011346	DQ861919
*Tribolium castaneum* (flour beetle)	AC154132	NW_001092869
*Tachypleus tridentatus* (horseshoe crab)	X53789	-----
*Ascaris lumbricoides* (nematode)	L22252	L22250
*Caenorhabditis elegans* (nematode)	X07829	X07828
*Schistosoma mansoni* (trematode)	L25920	-----
*Taenia solium* (tapeworm)	AF529186	-----
*Lytechinus variegatus* (sea urchin)	-----	U37266
*Strongylocentrotus purpuratus* (sea urchin)	X76389	NW_001323459
**Fungi, Ascomycota**		
*Saccharomyces cerevisiae* (budding yeast)	X12565	Siliciano *et al.* (1987)
*Schizosaccharomyces pombe* (fission yeast)	X14196	X15491
*Kluyveromyces lactis*	NC_006042	Guthrie & Patterson (1988)
*Candida albicans*	EU144231	EU144229
*Vanderwaltozyma polyspora*	NZ_AAZN01000268	-----
*Ashbya gossypii*	NC_005788	-----
**Fungi, Basidiomycota**		
*Erythrobasidium hasegawianum*	Tani & Ohshima (1991)	D63682
*Puccinia graminis*	AAWC01000866	-----
*Coprinopsis cinerea*	AACS01000244	-----
*Phanerochaete chrysosporium*	AADS01000210	-----
**Amoebozoa, Mycetozoa**		
*Dictyostelium discoideum *(slime mold)	AY953942	AY918063
*Physarum polycephalum* (slime mold)	-----	X13840
**Amoebozoa, Conosa**		
*Entamoeba histolytica*	U43841	BK006131
**Viridiplantae, Eudicot**		
*Arabidopsis thaliana* (thale cress)	X52527	X67145
*Vicia faba* (broad bean)	Solymosy & Pollak (1993)	Solymosy & Pollak (1993)
*Pisum sativum* (pea)	Solymosy & Pollak (1993)	X15933
*Solanum lycopersicum* (tomato)	X51447	-----
*Solanum tuberosum* (potato)	S83742	-----
*Populus trichocarpa* (Poplar)	NC_008469	NC_008470
**Viridiplantae, Monocot**		
*Oryza sativa* (rice)	NC_008405	DQ649301
*Triticum aestivum* (wheat)	X63066	-----
*Zea mays* (maize)	-----	Solymosy & Pollak (1993)
**Viridiplantae, Algae**		
*Chlamydomonas reinhardtii*	X71486	X71485
**Alveolata, Cilliophora**		
*Tetrahymena thermophila*	Orum *et al.* (1991)	Orum *et al.* (1991)
**Alveolata, Apicomplexa**		
*Plasmodium falciparum*	EF419774	EF140769
**Euglenozoa**		
*Trypanosoma brucei* (flagellate)	X57046	Solymosy & Pollak (1993)
*Crithidia fasciculata* (flagellate)	X78550	AF326336
*Leishmania tarentolae* (flagellate)	-----	X97621
*Leishmania mexicaca* (flagellate)	X82228	-----
*Leptomonas seymouri* (flagellate)	X78552	AJ245951
*Phytomonas* sp. (flagellate)	X82229	-----
